# 292 Augmented Renal Clearance in Burn Patients: Incidence and Discordance with Standard Creatinine Clearance Estimation

**DOI:** 10.1093/jbcr/irad045.267

**Published:** 2023-08-29

**Authors:** Scott W Mueller, Brittany Blass, Julia N Davis, Lyndsay A Deeter, Cameron Gibson, Amber D Kohler, Arek J Wiktor

**Affiliations:** University of Colorado Burn and Frostbite Center, Aurora, Colorado; University of Colorado School of Medicine, Aurora, Colorado; University of Colorado Skaggs School of Pharmacy and Pharmaceutical Sciences, Aurora, Colorado; University of Colorado School of Medicine, Aurora, Colorado; University of Colorado School of Medicine, Aurora, Colorado; University of Colorado School of Medicine, Aurora, Colorado; University of Colorado, Aurora, Colorado

## Abstract

**Introduction:**

Augmented renal clearance (ARC) is a phenomenon that occurs in some critically ill populations. ARC is associated with drug failure, commonly antimicrobials, due to subtherapeutic drug exposure. Patient specific factors for ARC have not been well described in the burn population. The aim of this study is to describe the incidence and patient characteristics associated with ARC in those admitted to a burn service for a mixed severity of illness.

**Methods:**

Consecutive adult patients admitted to an ABA verified burn center were screened under an institutional collaborative practice agreement for identification of ARC via a 12-hour urine collection (UC-12). Data was collected retrospectively under an IRB approved protocol. Creatinine clearance (CrCl) was calculated directly from UC-12 whereas Cockcroft-Gault (C-G) was calculated using a standard equation with adjusted body weight when applicable. A UC-12 CrCl greater than 130 mL/min was considered ARC and all values were compared to C-G estimations. Descriptive and inferential analyses were non-parametric using a two-sided alpha value of < 0.05 as significant.

**Results:**

A total of 53 patients over 8 months completed a UC-12 with a median 2 (range 1-9) collected during hospitalization. The population was largely male (81%), without trauma (91%), median age 43 (interquartile range [IQR] 35.5-58.5), total body surface area (TBSA) 20% (IQR 7.25-23.55), with thermal injuries (91%). Of the 53 patients, ARC was identified during hospitalization in 68%. There were no significant differences in median age, TBSA, proportion of trauma, or thermal burn between patients with ARC and those without (41 vs 48 years, 20.25 vs 13%, 11 vs 6%, and 94 vs 82%, respectively; p >0.1 for all). Of the 117 UC-12 measurements, ARC was identified in 61.5%, occurring a median 9 days (range 0-72) post injury. Overall median UC-12 CrCl was 148 mL/min (IQR 105-199) and C-G estimation was 139 mL/min (IQR 106-162) with a median discordance of 16 mL/min (IQR -14-58). Discordance of 30mL/min or more (positively or negatively) was frequent comparing actual CrCl to estimates using C-G (58%).

**Conclusions:**

ARC, as directly measured by UC-12, is common in a mixed burn population. Additional research is required to identify patients at increased risk for ARC and subsequent drug dosing strategies to maintain efficacy without undue risk of toxicity.

**Applicability of Research to Practice:**

Widely accepted estimations of CrCl may result in incorrect drug dosing and commonly underestimates actual CrCl which has implications for drug clearance, and therefore, dosing. Patients with ARC likely require dosing that exceeds FDA recommendations when titration to a dynamic effect or real-time pharmacokinetics are unavailable. Timed urine collection for CrCl calculation should be considered in the burn population.

